# Does early palliative identification improve the use of palliative care services?

**DOI:** 10.1371/journal.pone.0226597

**Published:** 2020-01-31

**Authors:** Nicole Mittmann, Ning Liu, Marnie MacKinnon, Soo Jin Seung, Nicole J. Look Hong, Craig C. Earle, Sharon Gradin, Saurabh Sati, Sandy Buchman, Ahmed Jakda, Frances C. Wright

**Affiliations:** 1 Cancer Care Ontario, Toronto, Ontario, Canada; 2 Sunnybrook Research Institute, Toronto, Ontario, Canada; 3 ICES, Toronto, Ontario, Canada; 4 Health Outcomes and PharmacoEconomics (HOPE) Research Centre, Toronto, Ontario, Canada; 5 Odette Cancer Centre, Toronto, Ontario, Canada; 6 Canadian Partnership Against Cancer, Toronto, Ontario, Canada; 7 Temmy Latner Centre for Palliative Care, Sinai Health System, Toronto, Ontario, Canada; 8 McMaster University, Hamilton, Ontario, Canada; 9 Ontario Palliative Care Network, Toronto, Ontario, Canada; 10 Grand River Regional Cancer Centre, Kitchener, Ontario, Canada; St. Michael’s Hospital, CANADA

## Abstract

**Purpose:**

To evaluate whether the early identification of patients who may benefit from palliative care impacts on the use of palliative, community and acute-based care services.

**Methods:**

Between 2014 and 2017, physicians from eight sites were encouraged to systematically identify patients who were likely to die within one year and would were thought to benefit from early palliative care. Patients in the INTEGRATE Intervention Group were 1:1 matched to controls selected from provincial healthcare administrative data using propensity score-matching. The use of palliative care, community-based care services (home care, physician home visit, and outpatient opioid use) and acute care (emergency department, hospitalization) was each evaluated within one year after the date of identification. The hazard ratio (HR) in the Intervention Group was calculated for each outcome.

**Results:**

Of the 1,185 patients in the Intervention Group, 951 (80.3%) used palliative care services during follow-up, compared to 739 (62.4%) among 1,185 patients in the Control Group [HR of 1.69 (95% CI 1.56 to 1.82)]. The Intervention Group also had higher proportions of patients who used home care [81.4% vs. 55.2%; HR 2.07 (95% CI 1.89 to 2.27)], had physician home visits [35.5% vs. 23.7%; HR 1.63 (95% CI 1.46 to 1.92)] or had increased outpatient opioid use [64.3% vs. 52.1%); HR 1.43 (95% CI 1.30 to 1.57]. The Intervention Group was also more likely to have a hospitalization that was not primarily focused on palliative care (1.42 (95% CI 1.28 to 1.58)) and an unplanned emergency department visit for non-palliative care purpose (1.47 (95% CI 1.32 to 1.64)).

**Conclusion:**

Physicians actively identifying patients who would benefit from palliative care resulted in increased use of palliative and community-based care services, but also increased use of acute care services.

## Background

Palliative care is focused on managing the physical, psychosocial, and spiritual needs of patients with life-threatening illnesses [1]. Early identification of patients who may benefit from a palliative approach to care has led to improved clinical outcomes, symptom control, quality of life and more efficient target use of health system resources across different populations of cancer patients [2–6].

Despite evidence of improved clinical outcomes for early identification of patients who would benefit form palliative care services, there is limited information on whether this early identification leads to earlier palliative care involvement and if so, how early in the trajectory. There is also limited information about the use of community-based care for such patients. Our objective was to examine the palliative, community-based services and acute services used by those actively identified as patients that would benefit from a palliative care services.

## Methods

### Study setting and the intervention

Cancer Care Ontario (CCO), the provincial cancer agency for Ontario, Canada, implemented the Integrating Early Palliative Care into Routine Practice for Patients with Cancer (INTEGRATE) study among four cancer centres and four primary care teams (2014–2017) (Appendix 1). The INTEGRATE study consisted of two interventions that included (i) inter-professional palliative care education [7]; and (ii) an integrated care model to facilitate early identification of patients who were likely to die within one year and would benefit from palliative care, early linkages to community-based resources for these patients, and improved communication between providers involved in patient care. The integrated care model was adapted from the Gold Standards Framework endorsed by the National Health System in the UK and developed by clinicians, allied health practitioners, administrators and patient and family advisors [8]. The overall goals of the INTEGRATE study were to: (i) enhance provider knowledge and confidence in palliative care delivery, (ii) identify patients who might benefit from palliative care earlier in their disease trajectory, (iii) increase the provision of palliative care and the use of palliative care tools, (iv) improve inter-professional collaboration and communication, and (v) improve the patient and caregiver experience. Health care providers asked the ‘Surprise Question’: “Would you be surprised if this patient were to die within 6–12 months?”[8]. If the answer was “no”, the patient was considered palliative care appropriate, and was included in the INTEGRATE study. Complete information on the methods is found in the original publication and presentation of the INTEGRATE study [9, 10]. The INTEGRATE study data collection period was a 1.5-years (January 2015-August 2016).

### Study design

We conducted a propensity score-matched study to evaluate the utilization of palliative services and community-based care between those in the INTEGRATE study (the Intervention Group) and comparable patients who were not enrolled in the INTEGRATE study (the Control Group), identified from population level administrative databases.

### Data sources

We used administrative databases housed and linked using unique encoded identifiers at ICES, Toronto, Ontario [11]. At ICES, the Registered Persons Database (RPDB) contains information about age, sex and postal code, as well as vital statistics data of all Ontario residents who have been issued a health card. Cancer diagnosis information came from the Ontario Cancer Registry (OCR), a database of information on all Ontario residents who have been diagnosed with cancer or who have died of cancer since 1964. The Canadian Institute for Health Information Discharge Abstract Database (CIHI-DAD) contains patient-level data for all inpatient care in hospitals in Ontario, including inpatient palliative care. The National Ambulatory Care Reporting System (NACRS) Database captures information on patient visits for hospital- and community-based ambulatory care, including emergency visits, same-day procedures, medical day care and cancer clinic care. The Ontario Health Insurance Plan (OHIP) Database contains billings from all physician services at both inpatient and outpatient settings. The Home Care Database (HCD) contains information on publicly funded healthcare services provided at private home by nurses, personal support workers, and other allied healthcare workers. The Ontario Drug Benefit (ODB) database contains claims for prescription drugs received under the Ontario Drug Benefit program, for Ontarians aged 65 years and above or those with financial difficulties. The Narcotics Monitoring System (NMS) collects data on dispensed prescriptions for narcotics, controlled substances and other monitored drugs, irrespective of whether the prescription is paid for under a publicly funded drug program, through private insurance, or by cash. The proportion and days in Alternate Level of Care (ALC) while hospitalized, namely patients occupying a bed in a hospital but do not require the intensity of resources or services provided in that setting, was also evaluated. The Activity Level Reporting (ALR) data constitute information on patient level activity within the cancer system focused on radiation and systemic therapy services [12].

### Exposure groups

A total of 1,196 patients were identified as possibly benefiting from palliative care using the Surprise Question across participating sites of the INTEGRATE study (Intervention Group). These patients were linked to the provincial health system administrative data via unique encoded patient identifiers. Excluded were those with missing demographic information on age, sex, and area of residence (N = 9), resulting in 1,187 patients remaining in the Intervention Group. The date when a patient in the INTEGRATE Intervention Group was identified defined his/her index date. Patients in the Intervention Group were followed from this index date to the earliest of 360 days thereafter; death date; date of last contact from administrative data; or the study end date, which was March 31, 2017. The Control Group was derived from the RPDB. Controls were patients who were not enrolled in the INTEGRATE study and were comparable with the Intervention Group in terms of all covariates assessed on the index date, as described below. For a Control Group patient who remained alive during the study period, the index date was defined as the date when the matched Intervention Group patient was identified. While for a Control Group patient who died during the study period, the index date was defined as his/her death date minus the same number of follow-up days of his/her matched Intervention patient.

### Study outcomes

The primary study outcomes were palliative healthcare utilization during the follow-up period. We used a previously published algorithm to identify palliative care services at both inpatient and outpatient settings [13]. In this analysis, palliative care services were defined as overall palliative health services, palliative physician encounters, community home care visits, and physician home visits. We also examined outpatient opioid utilization in the follow up period. Databases used are listed in the Appendix 2. Secondary outcomes included whether the death occurred at home using OHIP physician billing code of pronouncement of death in the home. As palliative-intent radiotherapy has been shown to be well-tolerated and cost-effective in managing symptoms and improving quality of life among incurable cancer patients [14], we further evaluated the utilization of palliative radiation in a subset of cancer patients who died before August 31, 2016. This subset was selected based on the availability of ALR radiation data. Finally, we conducted additional analyses examining the use of hospital-based services—unplanned emergency department visits, hospitalizations that were not for palliative care, intensive care unit (ICU) admissions and alternative level of care (ALC) that occurred during the follow-up period.

### Covariates

Factors that could potentially account for the difference in palliative care utilization between the Intervention and the Control groups were considered in the matching process. Patient socio-demographic factors included: patient age at the index date, area of residence denoted by Local Health Integration Network (LHIN), rurality, and neighborhood income quintile measured at dissemination area (DA) level. A DA is a standardized small, relatively stable geographic unit comprising a population of 400 to 700 persons of homogenous socio-economic status. Disease characteristics examined included: cancer status (y/n), and comorbidities, defined by the 29 Johns Hopkins Aggregated Diagnostic Groups (ADGs) [15]. For patients with cancer, we further considered the type of primary cancer and cancer stage. Previous healthcare utilization was measured as the resource utilization band (RUB). Both ADG and RUB were derived using the Johns Hopkins University’s Adjusted Clinical Group technique based on a patient’s age, gender and diagnostic information from both ambulatory and inpatient care settings (i.e., inpatient care, emergency department visits, and physician visits) in the two years prior to index date. This technique captures the specific clustering of morbidities experienced by a patient over the specified time period and takes into consideration the numerous conditions the patient develops and the pattern of these morbidities.

### Analysis

Matching was performed separately for the Intervention Group patients who remained alive, and who died during the follow-up. First, the propensity score (PS) method was used to match 1 patient who was alive in the Control Group to each Intervention Group patient (1:1 match) who remained alive during follow-up. The PS was estimated using a logistic regression model where being in the Intervention Group was regressed on patients’ demographics (age, LHIN of residence, rurality, and neighborhood income quintile), pre-existing comorbidities in the 2 years before the index date (denoted by the 29 ADGs), previous resource utilization pattern denoted by RUB, and cancer diagnosis and stage (if appropriate). A nearest-neighbour greedy matching algorithm was applied based on the PS, with a caliper width of 0.2 standard deviations of the logit of the PS [16]. Matching was done without replacement.

Then a separate PS model was used to select 1 deceased patient from the Control Group to match to each Intervention Group patient (1:1 match) who died during follow-up. In addition to all prior matching criteria, the matching of deceased patients also required that the death date of a control patient to be within 60 days from the death date of the matched Intervention Group patient.

After matching, standardized differences were used to compare all covariates between the Intervention Group and the Control Group, with an absolute value < 0.1 indicating good balance [17].

For each outcome, we reported the number and percentage of patients who experienced the outcome by group. For an outcome where frequencies of utilization could be quantified (i.e., palliative care visits, home care visits, and physician home visits), we further reported the number of visits per 360 patient days, calculated as the number of visits for a specific type of service during the follow-up period divided by the total number of patient days of the same period and multiplied by 360. To describe how quickly service utilization occurred, and to account for different length of follow-up between patients, the cumulative incidence function (CIF) was used to estimate the probability of having used the service in each month of follow-up, taking death as a competing event [18, 19]. For all outcomes except pronouncement of death in the home, a Fine and Gray subdistribution hazards model was employed to generate hazard ratios (HR) and 95% confidence intervals (CI) comparing the Intervention Group to the Control Group (the referent), taking death as a competing event [18, 19]. A subdistribution hazards model was used to estimate the HR for pronouncement of death in the home in the Intervention Group, taking death that occurred outside of a private home as a competing event. To account for matching, robust sandwich variance estimators were used to calculate the 95% CI of the HR [20, 21].

For the primary outcome, we performed stratified analysis by location of care (cancer centre vs. primary care), further evaluating the effect of intervention among patients with and without a cancer diagnosis. Chi-square tests were used to compare categorical covariates. For continuous covariates, t-tests compared means and Mann Whitney U test compared medians. All statistical analyses were performed using SAS for UNIX, Version 9.4 (SAS Institute, Cary, NC, USA). Statistical significance was deemed to be 0.05 for all comparisons.

Ethics approval for this secondary analysis was granted by the research ethics board of Sunnybrook Health Sciences Centre, Toronto, Canada.

## Results

### Demographics

Of the 1,187 patients remaining in the Intervention Group after exclusion, 1,185 (99.8%) were matched to a control from the province. The Intervention Group and the Control group were well-balanced on demographics, cancer diagnosis, comorbidities, previous health system resource utilization, and death status. The mean age was 70 years in both groups. Fifty-four percent (54.3%) of the linked and matched cohort were male. The majority of patients (76.2%) had a diagnosis of cancer recorded in OCR. The follow-up period was 230 days after the index date on average. In each group, 629 patients died during the follow-up period (Table 1).

**Table 1:** Characteristics of the INTEGRATE Intervention Group and their matched controls.

Characteristics	Measure	INTEGRATE Intervention group N = 1,185	Control N = 1,185	Standardized difference	P-Value
***At the index date***					
Sex	F	546 (46.1%)	537 (45.3%)	0.02	0.711
	M	639 (53.9%)	648 (54.7%)	0.02	
Age, yr	Mean ± SD	69.7 ± 12.8	70.1 ± 13.3	0.02	0.558
	Median (IQR)	70 (61–79)	71 (62–80)	0.04	0.37
Nearest Census Based Neighbourhood Income Quintile (within CMA/CA)	1—lowest	233 (19.7%)	236 (19.9%)	0.01	0.968
	2	232 (19.6%)	243 (20.5%)	0.02	
	3	228 (19.2%)	218 (18.4%)	0.02	
	4	238 (20.1%)	239 (20.2%)	0	
	5—highest	254 (21.4%)	249 (21.0%)	0.01	
Rural resident	Y	214 (18.1%)	214 (18.1%)	0	1
Local Health Integration Network (LHIN) of residence	Erie St. Clair	0 (0.0%)	<6	0.06	0.876
	South West	<6	<6	0.02	
	Waterloo Wellington	<6	<6	0	
	Hamilton Niagara Haldimand Brant	<6	10 (0.8%)	0.08	
	Central West	20 (1.7%)	23 (1.9%)	0.02	
	Mississauga Halton	19 (1.6%)	19 (1.6%)	0	
	Toronto Central	80 (6.8%)	78 (6.6%)	0.01	
	Central	109 (9.2%)	106 (8.9%)	0.01	
	Central East	79 (6.7%)	83 (7.0%)	0.01	
	South East	26 (2.2%)	26 (2.2%)	0	
	Champlain	600 (50.6%)	592 (50.0%)	0.01	
	North Simcoe Muskoka	236 (19.9%)	230 (19.4%)	0.01	
	North East	9 (0.8%)	10 (0.8%)	0.01	
	North West	0 (0.0%)	<6	0.04	
***Had a cancer diagnosis recorded in Ontario Cancer Registry***		903 (76.2%)	903 (76.2%)	0.00	1.000
***Pre-existing health problems and resource utilization in the 2 years before the Index date***					
Resource utilization band	0–3	212 (17.9%)	211 (17.8%)	0.00	0.352
	4	353 (29.8%)	323 (27.3%)	0.06	
	5	620 (52.3%)	651 (54.9%)	0.05	
Aggregated Diagnostic Groups (ADG*) score	Mean ± SD	9.1 ± 3.5	9.1 ± 3.4	0.01	0.732
	Median (IQR)	9 (7–11)	9 (7–11)	0.02	0.565
	0–5	188 (15.9%)	180 (15.2%)	0.02	0.861
	6–7	209 (17.6%)	214 (18.1%)	0.01	
	8–9	266 (22.4%)	250 (21.1%)	0.03	
	10–11	248 (20.9%)	265 (22.4%)	0.03	
	>=12	274 (23.1%)	276 (23.3%)	0	
Time Limited: Minor		352 (29.7%)	365 (30.8%)	0.02	0.561
Time Limited: Minor-Primary Infections		683 (57.6%)	667 (56.3%)	0.03	0.507
Time Limited: Major		414 (34.9%)	433 (36.5%)	0.03	0.415
Time Limited: Major-Primary Infections		263 (22.2%)	276 (23.3%)	0.03	0.524
Allergies		67 (5.7%)	47 (4.0%)	0.08	0.055
Asthma		109 (9.2%)	105 (8.9%)	0.01	0.774
Likely to Recur: Discrete		610 (51.5%)	595 (50.2%)	0.03	0.538
Likely to Recur: Discrete-Infections		319 (26.9%)	320 (27.0%)	0	0.963
Likely to Recur: Progressive		238 (20.1%)	254 (21.4%)	0.03	0.418
Chronic Medical: Stable		895 (75.5%)	930 (78.5%)	0.07	0.088
Chronic Medical: Unstable		709 (59.8%)	717 (60.5%)	0.01	0.737
Chronic Specialty: Stable-Orthopedic		33 (2.8%)	36 (3.0%)	0.02	0.714
Chronic Specialty: Stable-Ear, Nose, Throat		65 (5.5%)	63 (5.3%)	0.01	0.856
Chronic Specialty: Stable-Eye		220 (18.6%)	212 (17.9%)	0.02	0.67
Chronic Specialty: Unstable-Orthopedic		64 (5.4%)	55 (4.6%)	0.03	0.397
Chronic Specialty: Unstable-Ear, Nose, Throat		0	0	N/A	N/A
Chronic Specialty: Unstable-Eye		210 (17.7%)	195 (16.5%)	0.03	0.413
Dermatologic		252 (21.3%)	226 (19.1%)	0.05	0.183
Injuries/Adverse Effects: Minor		343 (28.9%)	352 (29.7%)	0.02	0.685
Injuries/Adverse Effects: Major		376 (31.7%)	389 (32.8%)	0.02	0.568
Psychosocial: Time Limited, Minor		105 (8.9%)	98 (8.3%)	0.02	0.607
Psychosocial: Recurrent or Persistent, Stable		370 (31.2%)	396 (33.4%)	0.05	0.253
Psychosocial: Recurrent or Persistent, Unstable		190 (16.0%)	217 (18.3%)	0.06	0.141
Signs/Symptoms: Minor		859 (72.5%)	879 (74.2%)	0.04	0.353
Signs/Symptoms: Uncertain		1,017 (85.8%)	1,003 (84.6%)	0.03	0.418
Signs/Symptoms: Major		995 (84.0%)	969 (81.8%)	0.06	0.156
Discretionary**		319 (26.9%)	328 (27.7%)	0.02	0.678
See and Reassure		92 (7.8%)	86 (7.3%)	0.02	0.640
Prevention/Administrative		614 (51.8%)	628 (53.0%)	0.02	0.565
***During follow-up***					
Days of follow-up	Mean ± SD	230.1 ± 127.1	230.1 ± 127.1	0.00	1.000
	Median (IQR)	260 (107–360)	260 (107–360)	0.00	1.000
Died during follow-up		629 (53.1%)	629 (53.1%)	0.00	1.000

* Conditions denoted by the Johns Hopkins Aggregated Diagnostic Groups (ADG), derived using diagnostic information in inpatient hospitalization, emergency department visits, and physician visits in the 2 years before the index date.

** The ACG system categorizes ICD-9 / ICD-10 diagnosis codes into one of 32 ADGs. These are two of those 32 ADGs and nomenclature directly outputted from the software, like all others ranging from “Time Limited: Minor” to “Prevention/Administrative” in this table. Examples of “discretionary”: inguinal hernia, sebaceous cyst. Examples of “See and reassure”: Hypertrophy of breast, localized adiposity.

### Palliative resource use

**Fig 1:**
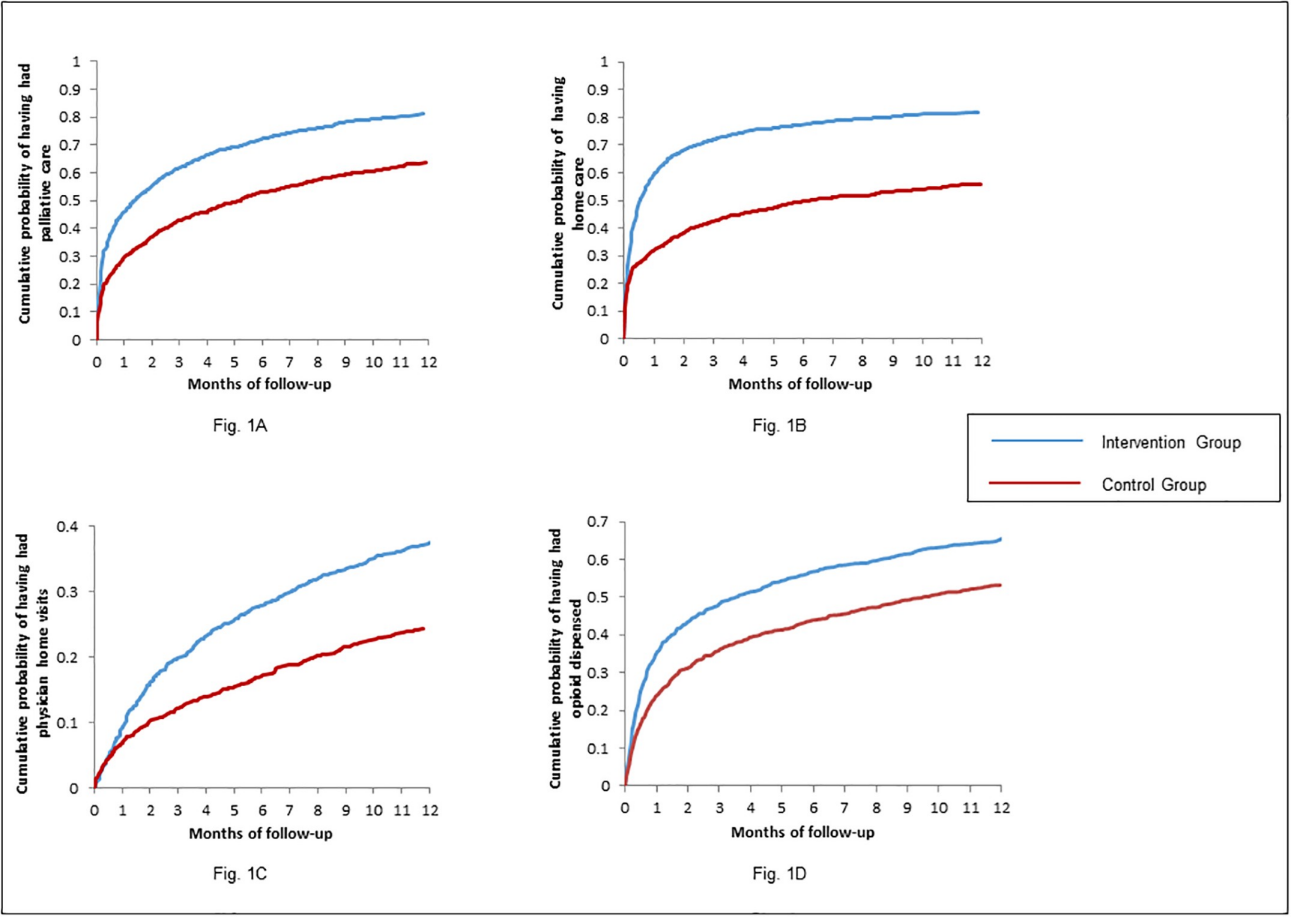
Cumulative probability curves. (A) Cumulative probability of having palliative care during follow-up, with death as a competing event. (B) Cumulative probability of having a home care visit during follow-up, with death as a competing event. (C) Cumulative probability of having a physician home visit during follow-up, with death as a competing event. (D) Cumulative probability of using opioid as an outpatient during follow-up, with death as a competing event.

**Fig 2:**
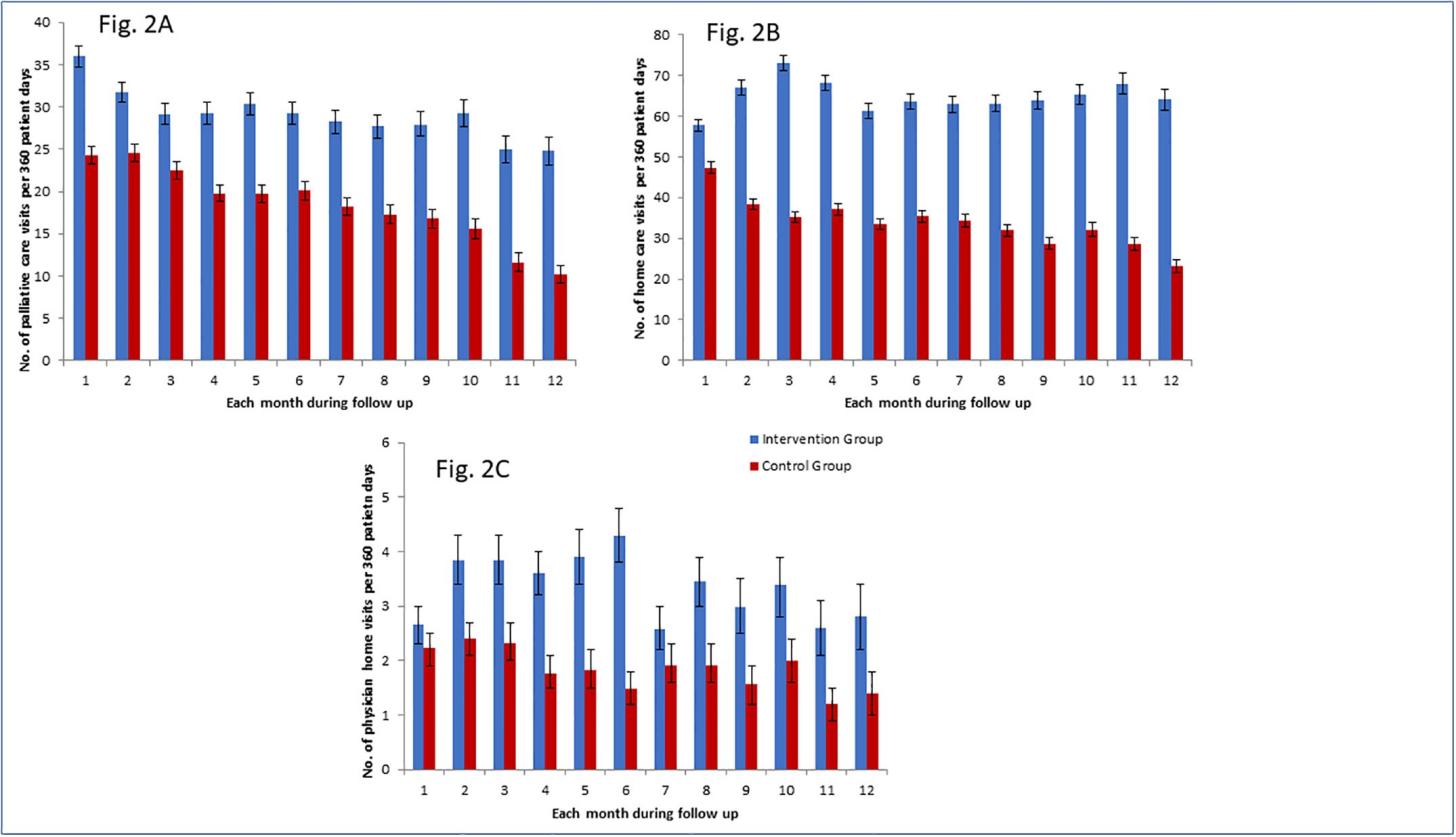
Number of resources used. (A) Number of palliative care visits per 360 patient days during each month of follow-up (B) Number of home care visits per 360 patient days during each month of follow-up (C) Number of physician home visits per 360 patient days during each month of follow-up.

Of the 1,185 patients in the Intervention Group, 951 (80.3%) used one or more palliative care services during the follow-up period, in contrast to 739 (62.4%) among patients in the Control Group. The cumulative probability of receiving palliative health services in the Intervention Group and the Control Group was 61.9% vs.43.0% at 3 months, 72.2% vs. 53.0% at 6 months, and 81.3% vs. 63.5% at 12 months of follow-up, respectively (Fig 1A), with a resultant HR of 1.69 (95% CI 1.56 to 1.82) (Table 2). The rate of palliative care visits was higher in the Intervention Group than that in the Control Group over the entire follow-up period (29.7 vs. 19.6 visits per 360 patient days) (Table 2), and in each month of follow-up (Fig 2A). Compared to the Control Group, the proportion of primary care and cancer patients who used community-based home care [Hazard Ratio 2.07 (95% CI 1.89 to 2.27)], had a physician home visit [HR: 1.63 (95% CI 1.46 to 1.92)], and used opioid as an outpatient [HR: 1.43 (95% CI 1.30 to 1.57)] was each statistically higher in the Intervention Group. The Intervention Group had more ALC days [HR 1.57, (95% CI 1.22 to 2.00)] (Table 2). Both the cumulative probability of using each service (Fig 1B, 1C and 1D), and the rate of service utilization per 360 days (Fig 2B and 2C) were higher in the Intervention Group, in each month during follow-up. The results persisted after stratifying by location of palliative identification (cancer clinic [Appendix 3] and primary care [Appendix 4]).

**Table 2:** Utilization of palliative care service and community-based services during the follow-up period, between the INTEGRATE group and the matched Control Group.

Outcomes	INTEGRATE Intervention Group N = 1,185	Control Group N = 1,185
Palliative care		
N (%) used palliative care	951 (80.3)	739 (62.4)
Number of visits per 360 patient days (95% CI)	29.7 (29.4 to 30.1)	19.6 (19.3 to 19.9)
Hazard Ratio (95% CI) *	1.69 (1.56 to 1.82)	1.00 (Referent)
Home care		
N (%) had a home care visit	964 (81.4)	654 (55.2)
Number of visits per 360 patient days (95% CI)	64.7 (64.2 to 65.3)	35.3 (34.9 to 35.7)
Hazard Ratio (95% CI) *	2.07 (1.89 to 2.27)	1.00 (Referent)
Physician home visit		
N (%) had a physician home visit	432 (36.5)	281 (23.7)
Number of visits per 360 patient days (95% CI)	3.4 (3.3 to 3.5)	1.9 (1.8 to 2.0)
Hazard Ratio (95% CI) *	1.67 (1.46 to 1.92)	1.00 (Referent)
Outpatient opioid use		
N (%) had any outpatient opioid dispensed	762 (64.3)	617 (52.1)
Hazard Ratio (95% CI) *	1.43 (1.30 to 1.57)	1.00 (Referent)
Alternative Level of Care		
N (%) designated Alternative Level of Care	153 (12.9)	100 (8.4)
Hazard Ratio (95% CI) *	1.57 (1.22 to 2.00)	1.00 (Referent)
Pronouncement of death in the home (N = 629)‡		
N (%) pronounced death in the home	99 (15.7)	76 (12.1)
Hazard Ratio (95% CI) †	1.32 (0.98 to 1.79)	1.00 (Referent)
Palliative radiation (N = 364)§		
N (%) used palliative radiation	158 (43.4)	84 (23.1)
Hazard Ratio (95% CI) *	2.34 (1.85 to 2.97)	1.00 (Referent)

*: Based on Fine and Gray subdistribution hazard model, taking death as a competing event. Robust sandwich variance estimates were used to account for matched pairs.

^†^: Based on Fine and Gray subdistribution hazard model, taking death at other places as a competing event. Robust sandwich variance estimates were used to account for matched pairs.

^‡^: Among patients who died during the follow-up period.

^§^: Among cancer patients who died before August 31, 2016.

### Secondary analyses

Of the 629 matched pairs who died during follow-up, 99 (15.7%) in the Intervention Group, and 76 (12.1%) in the Control Group were pronounced dead at home [HR: 1.32 (95% CI 0.98 to 1.79)]. In the 364 pairs of cancer patients who died before August 31, 2016, the proportion of patients who had received palliative radiation was 43.4% in the Intervention group, and 23.1% in the Control Group [HR of 2.34 (95% CI 1.85 to 2.97)] (Table 2).

The Intervention Group was also more likely to have a hospitalization [HR 1.42, (95% CI 1.28 to 1.58)] and an emergency department visit [HR 1.47, (95% CI 1.32 to 1.64)] with mostly non-palliative intent recorded. The two groups, however, did not differ in ICU admission [HR 0.98, (95% CI 0.76 to 1.19)] but the INTEGRATE group had shorter lengths of ICU stay (Appendix 5). In Appendix 6, of the 1,258 patients (629 matched pairs) who died during follow-up, 1,110 (88.2%) had information on cause of death. The proportion of patients with unknown cause of death was comparable between the Intervention and the Control Group (11.1% vs. 12.4%). In both groups, the majority of patients died of cancer (78.6% vs. 67.6%), followed by cardio vascular disease (3.5% vs. 7.6%) and respiratory disease (2.4% vs. 4.9%). The proportion died of unexpected causes such as injury, poison, and external causes was very low, and there was no meaningful difference in such causes between two groups. In fact, these numbers were too low to be reported according to ICES Data Policy, which stipulates that no small cells (i.e., < 6 observations) can be reported. In Appendix 7, the comparison of baseline characteristics between the Intervention Group vs. the Control Group, among those who died. The two groups were well-balanced on all baseline characteristics, except that the Intervention Group was on average 1 year younger (70 y vs. 71 y) and had higher proportion of patients who had allergy (6.4% vs. 3.3%). Appendix 8 provides baseline characteristics of patients who remained alive by the end of follow-up period in the Intervention Group and the matched Control Group and were well balanced amongst patient characteristics. Supporting tables provide additional information on the participating sites (S1 Table), codes used to define the baseline characteristics and outcomes (S2 Table) and utilization of resources (S3–S5 Tables) and characteristics of patients who were alive and those who died in both the intervention and control groups (S6–S8 Tables).

## Discussion

In this propensity-score matched cohort study of patients who were likely to die within one year, we found that actively identifying patients who may benefit from a palliative care approach increased the utilization of palliative care services and community-based care such as home care, physician home visits, and outpatient opioid use. This effect was observed with identification both in a cancer clinic setting and a primary care setting. This effect was also seen in very complex cancer patients including those with lung cancer and glioblastoma. There was also an increase in acute care utilization, namely emergency department encounters and hospitalizations for the intervention group.

Earlier access to palliative care is in line with quality standards for quality care in the province of Ontario [22]. Nevertheless, the most recent data from the Canadian Institute for Health Information shows that in Ontario and Alberta, fewer than 15% receive palliative care at home [23]. Our results are also congruent with other end of life studies. Tanuseputro and colleagues showed that less than one-third of the population receive end-of-life home care or a physician visit in their last year of life [13]. Another study evaluating the Gold Standards Framework in Care Homes in the UK found that the Framework helps to improve the quality and quantity of communication, coordination and collaboration between nursing home staff and other clinicians [24].

The demonstrated increase in palliative use occurred in the absence of any formal system changes or interventions. We propose that the increased resource utilization of palliative services in the Intervention Group was due to improved education and communication between patients, families and health care providers. We hypothesize that system changes or formal interventions would further increase palliative resource use.

Despite the increased palliative health system resource use found in the intervention group, we also found an increase in ED visits and hospitalizations. These increased visits may be related to a number of factors including symptoms, complications and medications changes. We speculate that these increased visits might be the result of being more connected to the health system once identified as palliative or by being formally involved as study participants which could improve communication about when to use acute health services. Our results of increased ED visits concur with another study where increased ED visits in patients receiving home care has been reported. Investigators found that patients who received home care nursing visits were more likely to visit the emergency department during the event [25]. Our results also showed that INTEGRATE cases had more alternate level of care designations than controls, indicating that patients were occupying a bed in a hospital without actually requiring that intensity of health system resources. Another area for potential improvement in the health care system is around the extent of supports in the community or availability of hospice beds.

Although we examined health services utilization, we were not able to examine the effect of early palliative identification on quality of life in this study because we used administrative data that was encounter based not outcome based. A systematic review in advanced lung cancer patients found that those who received early palliative care had better quality of life and survival [26]. In a randomized controlled design, Temel and colleagues reported that newly diagnosed patients with metastatic non-small-cell lung cancer who received either early palliative care integrated with standard oncology care or standard oncologic care alone had significantly better quality of life and fewer depressive symptoms than those who received standard care [4]. These results were based on a randomized prospective design and not administrative databases.

Examination of real-world evidence for outcomes would help underscore these results. Similarly, another study on early versus delayed entry to concurrent palliative oncology care found significant improvement in one-year survival, quality of life and mood among those who received palliative care early [27]. In that study, there was no significant difference in resource use between the two groups in terms of hospital days, intensive care days, emergency room visits and chemotherapy, although this may be attributed to a lack of statistical power, multiple comparisons and disease cohort [28].

Our study has many strengths, such as linkage to actual provincial real-world resource utilization. Unlike previous studies, which focused on one centre or a few centres as part of a clinical trial, the INTEGRATE study is a real-world assessment of health outcomes when patients are identified early for involvement of palliative care are identified within a health care system. The improvements shown in access to palliative healthcare services were achieved without additional system support, funding and programs (apart from those already available within the health care system). Additional system supports such as access to 24–7 palliative care and advice in situations of crisis for both providers in the home and in the community, and patients and caregivers, as well as the presence of interdisciplinary teams with well-established information sharing and communication mechanisms in different settings [29–32] may improve the findings of this work.

In terms of limitations, first and foremost, we recognize that these results are not based on a randomized controlled clinical trial design and that participants were not randomly assigned to the INTEGRATE study. As such, the results could be subject to selection bias. However, our matching technique provided excellent matching results showing that the two cohorts were very similar. But it is possible that administrative data may not hold all potential matchable criteria and that unrecognized selection bias still exists. Moreover, for the hospitalization data, and ALC in particular, we don’t know the start and stop dates which means that we know the event happened but not exactly when the event happened. We are assuming that if a hospitalization had acute and ALC days that ALC happened after the acute period. Despite the aforementioned limitations, this observational comparative study suggests, that from a public payer perspective, early identification of patients that may benefit from palliative care services resulted in both more access and earlier access to palliative health services.

## Conclusions

Physicians actively identifying patients who would benefit from palliative care resulted in increased use of palliative care services an improved access to those services but had more hospital encounters. Earlier palliative care has been shown to improve quality of life and survival. Our results show that early identification led to an observed increase in access to palliative care services. Future work will focus on reasons for increased health service resource utilization and sequencing of events as well as focus on the cost of end of life care.

## Supporting information

S1 Table INTEGRATE pilot project participating sites. (DOCX)

S2 Table Datasets and specific codes used to define patient baseline characteristics and outcome variables. (DOCX)

S3 Table Utilization of palliative care service and community-based services during the follow-up period, between patients in the INTEGRATE Intervention Group who were identified in a cancer clinic setting and their matched Control Group. (DOCX)

S4 Table Utilization of palliative care service and community-based services during the follow-up period, between patients in the INTEGRATE Intervention Group who were identified in a primary care setting and their matched Control Group. (DOCX)

S5 Table Utilization of hospital-based services during the follow-up period, between the INTEGRATE Intervention Group and the matched Control Group. (DOCX)

S6 Table Cause of death among deceased patients, comparing the Intervention Group vs. the Control group. (DOCX)

S7 Table Baseline characteristics of deceased patients in the Intervention Group and the Control Group. (DOCX)

S8 Table Baseline characteristics of patients who remained alive by the end of follow-up period in the Intervention Group and the matched Control Group. (DOCX)
