# Supplementary material for: Does early palliative identification improve the use of palliative care services?
Source: PLoS One. 2020 Jan 31;15(1):e0226597. doi: 10.1371/journal.pone.0226597 (PMC6994244; doi:10.1371/journal.pone.0226597)
Supplement: S1 Table — (DOCX) [file pone.0226597.s001.docx]

S1 Table. INTEGRATE Pilot Project Participating Sites.

| **Integrated Models** | | **Toronto Central North** | **Toronto Central South** | **Champlain** | **North Simcoe Muskoka** |
| --- | --- | --- | --- | --- | --- |
| **Primary Care Model** | Institution | Sunnybrook Academic Family Health Team | Forest Hill Family Health Group | Petawawa Centennial Family Health Centre | Barrie and Community Family Health Team |
| **Cancer Centre Model** | Institution | Sunnybrook Health Sciences Centre | Princess Margaret Hospital | The Ottawa Hospital | The Royal Victoria Hospital |
|  | Disease Site | 1. CNS MCC & Clinic | 1. CNS MCC | 1. Lung DAP 2. Head and Neck Clinic | 1. Lung MCC 2. GI MCC |

DAP: Diagnostic Assessment Program. DAPs are a rapid-access strategy for patients to receive comprehensive diagnostic services through a single point of access, including multidisciplinary consultative expertise and patient information resources (CCO, 2017).

MCC: Multi-disciplinary Cancer Conference. MCCs are regularly scheduled meetings where healthcare providers discuss the diagnosis and treatment of individual cancer patients (CCO, 2010).

MDC: Multi-disciplinary clinic. MDCs co-locate multiple providers from different medical specialties to assess a patient together in an out- patient clinic setting.
