# Supplementary material for: Does early palliative identification improve the use of palliative care services?
Source: PLoS One. 2020 Jan 31;15(1):e0226597. doi: 10.1371/journal.pone.0226597 (PMC6994244; doi:10.1371/journal.pone.0226597)
Supplement: S2 Table — (DOCX) [file pone.0226597.s002.docx]

**S2 Table. Datasets and specific codes used to define patient baseline characteristics and outcome variables.**

| **Assessment** | **Variable** | **Data source and codes** |
| --- | --- | --- |
| Study outcomes | Palliative care | Ontario Health Insurance Plan (OHIP) physician billing codes: A945, B966, B997, B998, C882, C945, C982, G511, G512, W872, W882, W972, W982, K023, K700, C122, C123, K121, A180, K374, K735, E083.  Canadian Institute for Health Information Discharge Abstract Database (CIHI-DAD): dx10code = Z515, or prvserv = 00121, or patserv = 58, or inserv = 00121.  National Ambulatory Care Reporting System (NACRS): consultserv = 00121, or prvserv = 00121. |
|  | Home care | Any record in Home Care Database |
|  | Physician home visit | OHIP physician billing codes: A900, A901, A902, B960, B961, B962, B963, B964, B966, B990, B992, B993, B994, B996, B998 |
|  | Outpatient opioid | Ontario Drug Benefit (ODB) and Narcotics Monitoring System (NMS). Drug Identification Number (DIN) available upon request. |
|  | Pronouncement of death at home | OHIP physician billing codes: A902 |
|  | Palliative radiation | Radiation dataset in Activity Level Reporting (ALR): intent_of_radiation_treatment = P |
|  | Hospitalization that was not mainly for palliative care | Any records in CIHI DAD except those where dx10code 1 = Z515 or patserv = 58 |
|  | Number of hospital days | Calculated using admdate and ddate in CIHI DAD and required days to fall within the follow-up period. |
|  | Intensive care unit admission (ICU) admission | If a patient had any ICU days during the follow-up period, determined using scuadmdate and scuddate in CIHI DAD. |
|  | Number of ICU days | Calculated using scuadmdate and scuddate in CIHI DAD and required days to fall within the follow-up period. |
| Patient characteristics | Sex | Registered Persons Database (RPDB) |
|  | Age on index date | RPDB |
|  | Rural or urban residence | RPDB |
|  | Residential income | RPDB |
|  | Local Health Integration Network (LHIN) of residence | RPDB |
|  | Diagnosis of cancer | Any malignant neoplasm recorded in Ontario Cancer Registry |
|  | Pre-existing health problems in the 2 years before index date, denoted by 29 Johns Hopkins Aggregated Diagnostic Groups | All ICD-9 and ICD-10 diagnosis codes identified from CIHI DAD, NACRS and OHIP in the 2 years before index date were supplied to the Johns Hopkins ACG System |
|  | Previous resource utilization in the 2 years before index date | All ICD-9 and ICD-10 diagnosis codes identified from CIHI DAD, NACRS and OHIP in the 2 years before index date were supplied to the Johns Hopkins ACG System |
|  | Death during follow-up | RPDB |
