# Supplementary material for: Does early palliative identification improve the use of palliative care services?
Source: PLoS One. 2020 Jan 31;15(1):e0226597. doi: 10.1371/journal.pone.0226597 (PMC6994244; doi:10.1371/journal.pone.0226597)
Supplement: S3 Table — (DOCX) [file pone.0226597.s003.docx]

**S3 Table. Utilization of palliative care service and community-based services during the follow-up period, between patients in the INTEGRATE Intervention Group who were identified in a cancer clinic setting and their matched Control Group.**

| **Outcomes** | **INTEGRATE**  **Intervention Group N=905** | **Control Group**  **N=905** |
| --- | --- | --- |
| Palliative care |  |  |
| N (%) used palliative care | 763 (84.3) | 617 (68.2) |
| Number of visits per 360 patient days (95% CI) | 34.9 (34.4 to 35.4) | 23.6 (23.2 to 24.0) |
| Hazard Ratio (95% CI) * | 1.63 (1.49 to 1.78) | 1.00 (Referent) |
| Home care |  |  |
| N (%) used home care | 769 (85.0) | 535 (59.1) |
| Number of visits per 360 patient days (95% CI) | 64.9 (64.3 to 65.6) | 36.2 (35.7 to 36.7) |
| Hazard Ratio (95% CI) * | 2.08 (1.87 to 2.31) | 1.00 (Referent) |
| Physician home visit |  |  |
| N (%) had a physician home visit | 326 (36.0) | 234 (25.9) |
| Number of visits per 360 patient days (95% CI) | 3.7 (3.5 to 3.8) | 2.3 (2.1 to 2.4) |
| Hazard Ratio (95% CI) * | 1.49 (1.28 to 1.73) | 1.00 (Referent) |
| Outpatient opioid use |  |  |
| N (%) had any outpatient opioid dispensed | 606 (67.0) | 504 (56.0) |
| Hazard Ratio (95% CI) * | 1.40 (1.26 to 1.55) | 1.00 (Referent) |

*: Based on Fine and Gray subdistribution hazard model, taking death as a competing event. Robust sandwich variance estimates were used to account for matched pairs.
