# Supplementary material for: Does early palliative identification improve the use of palliative care services?
Source: PLoS One. 2020 Jan 31;15(1):e0226597. doi: 10.1371/journal.pone.0226597 (PMC6994244; doi:10.1371/journal.pone.0226597)
Supplement: S4 Table — (DOCX) [file pone.0226597.s004.docx]

**S4 Table. Utilization of palliative care service and community-based services during the follow-up period, between patients in the INTEGRATE Intervention Group who were identified in a primary care setting and their matched Control Group.**

| **Outcomes** | **INTEGRATE**  **Intervention Group N=280** | **Control Group**  **N=280** |
| --- | --- | --- |
| Palliative care |  |  |
| N (%) used palliative care | 188 (67.1) | 122 (43.6) |
| Number of visits per 360 patient days (95% CI) | 16.0 (15.5 to 16.6) | 8.9 (8.5 to 9.3) |
| Hazard Ratio (95% CI) * | 2.01 (1.68 to 2.41) | 1.00 (Referent) |
| Home care |  |  |
| N (%) used home care | 195 (69.6) | 119 (42.5) |
| Number of visits per 360 patient days (95% CI) | 64.3 (63.2 to 65.4) | 32.9 (32.1 to 33.6) |
| Hazard Ratio (95% CI) * | 2.16 (1.75 to 2.67) | 1.00 (Referent) |
| Physician home visit |  |  |
| N (%) had a physician home visit | 106 (37.9) | 47 (30.7) |
| Number of visits per 360 patient days (95% CI) | 2.6 (2.4 to 2.8) | 1.0 (0.9 to 1.1) |
| Hazard Ratio (95% CI) * | 2.58 (1.85 to 3.59) | 1.00 (Referent) |
| Outpatient opioid use |  |  |
| N (%) had any outpatient opioid dispensed | 156 (55.7) | 113 (40.4) |
| Hazard Ratio (95% CI) * | 1.60 (1.29 to 1.97) | 1.00 (Referent) |

*: Based on Fine and Gray subdistribution hazard model, taking death as a competing event. Robust sandwich variance estimates were used to account for matched pairs.
