# Supplementary material for: Does early palliative identification improve the use of palliative care services?
Source: PLoS One. 2020 Jan 31;15(1):e0226597. doi: 10.1371/journal.pone.0226597 (PMC6994244; doi:10.1371/journal.pone.0226597)
Supplement: S5 Table — (DOCX) [file pone.0226597.s005.docx]

**S5 Table. Utilization of hospital-based services during the follow-up period, between the INTEGRATE Intervention Group and the matched Control Group.**

| **Outcomes** | **INTEGRATE**  **Intervention Group N=1,185** | **Control Group**  **N=1,185** |
| --- | --- | --- |
| Hospitalization that was not mainly for palliative care |  |  |
| N (%) had a hospitalization | 380 (42.0) | 291 (32.2) |
| Number of hospital days per 360 patient days (95% CI) | 15.7 (15.4 to 16.0) | 12.3 (12.0 to 12.5) |
| Hazard Ratio (95% CI) * | 1.42 (1.28 to 1.58) | 1.00 (Referent) |
| Intensive care unit (ICU) |  |  |
| N (%) admitted to ICU | 149 (12.6) | 157 (13.2) |
| Number of ICU days per 360 patient days (95% CI) | 0.8 (0.7 to 0.8) | 1.0 (0.9 to 1.1) |
| Hazard Ratio (95% CI) * | 0.98 (0.76 to 1.19) | 1.00 (Referent) |
| Unplanned emergency department (ED) visit |  |  |
| N (%) had a ED visit | 633 (53.4) | 483 (40.8) |
| Number of visits per 360 patient days (95% CI) | 1.19 (1.8 to 2.0) | 1.3 (1.3 to 1.4) |
| Hazard Ratio (95% CI) * | 1.47 (1.32 to 1.64) | 1.00 (Referent) |

*: Based on Fine and Gray subdistribution hazard model, taking death as a competing event. Robust sandwich variance estimates were used to account for matched pairs.
