# Supplementary material for: Does early palliative identification improve the use of palliative care services?
Source: PLoS One. 2020 Jan 31;15(1):e0226597. doi: 10.1371/journal.pone.0226597 (PMC6994244; doi:10.1371/journal.pone.0226597)
Supplement: S6 Table — (DOCX) [file pone.0226597.s006.docx]

**S6 Table. Cause of death among deceased patients, comparing the Intervention Group vs. the Control group.**

| **Cause of death** | **Intervention Group** | **Control Group** | **Standardized difference** |
| --- | --- | --- | --- |
|  | **N=629** | **N=629** |  |
| Cancer | 492 (78.2%) | 425 (67.6%) | 0.24 |
| Cardio vascular disease | 22 (3.5%) | 48 (7.6%) | 0.18 |
| Respiratory disease | 15 (2.4%) | 31 (4.9%) | 0.14 |
| Mental illness | 12 (1.9%) | 9 (1.4%) | 0.04 |
| Disease of digestive system | < 6 | 9 (1.4%) | 0.10 |
| Immune or endocrine disease | < 6 | < 6 | 0.00 |
| Disease of the nervous system | < 6 | 6 (1.0%) | 0.11 |
| Infection | < 6 | < 6 | 0.08 |
| Injury, or poison by an aesthetics and therapeutic gases | < 6 | < 6 | 0.09 |
| External causes of mortality such as fall, overexertion or accident | < 6 | < 6 | 0.02 |
| Other causes | < 6 | < 6 | 0.02 |
| Unknown cause of death | 70 (11.1%) | 78 (12.4%) | 0.04 |
