# Supplementary material for: Does early palliative identification improve the use of palliative care services?
Source: PLoS One. 2020 Jan 31;15(1):e0226597. doi: 10.1371/journal.pone.0226597 (PMC6994244; doi:10.1371/journal.pone.0226597)
Supplement: S7 Table — (DOCX) [file pone.0226597.s007.docx]

**S7 Table. Baseline characteristics of deceased patients in the Intervention Group and the Control Group**

| **Variable** | **Value** | **Intervention Group**  **N=629** | **Control Group**  **N=629** | **Standardized difference** |
| --- | --- | --- | --- | --- |
| Sex | F | 283 (45.0%) | 284 (45.2%) | 0 |
|  | M | 346 (55.0%) | 345 (54.8%) | 0 |
| Age | Mean ± SD | 69.8 ± 11.7 | 71.3 ± 11.6 | 0.12 |
|  | Median (IQR) | 70 (62-78) | 71 (63-79) | 0.12 |
| Income quintile | 1-lowest | 122 (19.4%) | 130 (20.7%) | 0.03 |
|  | 2 | 130 (20.7%) | 136 (21.6%) | 0.02 |
|  | 3 | 116 (18.4%) | 104 (16.5%) | 0.05 |
|  | 4 | 135 (21.5%) | 131 (20.8%) | 0.02 |
|  | 5-highest | 126 (20.0%) | 128 (20.3%) | 0.01 |
| Rural resident | Y | 119 (18.9%) | 109 (17.3%) | 0.04 |
| Local Health Integration Network (LHIN) of residence | Erie St. Clair | <6 | <6 | 0.08 |
|  | South West | <6 | <6 | 0.03 |
|  | Waterloo Wellington | <6 | <6 | 0 |
|  | Hamilton Niagara Haldimand Brant | <6 | 7 (1.1%) | 0.15 |
|  | Central West | 10 (1.6%) | 13 (2.1%) | 0.04 |
|  | Mississauga Halton | 11 (1.7%) | 11 (1.7%) | 0 |
|  | Toronto Central | 43 (6.8%) | 41 (6.5%) | 0.01 |
|  | Central | 58 (9.2%) | 55 (8.7%) | 0.02 |
|  | Central East | 40 (6.4%) | 44 (7.0%) | 0.03 |
|  | South East | 13 (2.1%) | 13 (2.1%) | 0 |
|  | Champlain | 322 (51.2%) | 314 (49.9%) | 0.03 |
|  | North Simcoe Muskoka | 121 (19.2%) | 115 (18.3%) | 0.02 |
|  | North East | 8 (1.3%) | 9 (1.4%) | 0.01 |
|  | North West | <6 | <6 | 0.06 |
| ***Pre-existing health problems and resource utilization in the 2 years before the Index date*** |  |  |  |  |
| Resource utilization band | 0-3 | 89 (14.1%) | 85 (13.5%) | 0.02 |
|  | 4 | 182 (28.9%) | 174 (27.7%) | 0.03 |
|  | 5 | 358 (56.9%) | 370 (58.8%) | 0.04 |
| Aggregated Diagnostic Groups (ADG) score | Mean ± SD | 9.4 ± 3.5 | 9.4 ± 3.4 | 0.01 |
|  | Median (IQR) | 9 (7-12) | 9 (7-12) | 0.02 |
|  | 0-5 | 87 (13.8%) | 80 (12.7%) | 0.03 |
|  | 6-7 | 104 (16.5%) | 111 (17.6%) | 0.03 |
|  | 8-9 | 143 (22.7%) | 139 (22.1%) | 0.02 |
|  | 10-11 | 136 (21.6%) | 140 (22.3%) | 0.02 |
|  | >=12 | 159 (25.3%) | 159 (25.3%) | 0 |
| Time Limited: Minor |  | 191 (30.4%) | 201 (32.0%) | 0.03 |
| Time Limited: Minor-Primary Infections |  | 386 (61.4%) | 373 (59.3%) | 0.04 |
| Time Limited: Major |  | 248 (39.4%) | 250 (39.7%) | 0.01 |
| Time Limited: Major-Primary Infections |  | 152 (24.2%) | 159 (25.3%) | 0.03 |
| Allergies |  | 40 (6.4%) | 21 (3.3%) | 0.14 |
| Asthma |  | 49 (7.8%) | 46 (7.3%) | 0.02 |
| Likely to Recur: Discrete |  | 327 (52.0%) | 320 (50.9%) | 0.02 |
| Likely to Recur: Discrete-Infections |  | 186 (29.6%) | 183 (29.1%) | 0.01 |
| Likely to Recur: Progressive |  | 125 (19.9%) | 142 (22.6%) | 0.07 |
| Chronic Medical: Stable |  | 485 (77.1%) | 490 (77.9%) | 0.02 |
| Chronic Medical: Unstable |  | 379 (60.3%) | 396 (63.0%) | 0.06 |
| Chronic Specialty: Stable-Orthopedic |  | 18 (2.9%) | 19 (3.0%) | 0.01 |
| Chronic Specialty: Stable-Ear,Nose,Throat |  | 33 (5.2%) | 36 (5.7%) | 0.02 |
| Chronic Specialty: Stable-Eye |  | 106 (16.9%) | 110 (17.5%) | 0.02 |
| Chronic Specialty: Unstable-Orthopedic |  | 35 (5.6%) | 24 (3.8%) | 0.08 |
| Chronic Specialty: Unstable-Ear,Nose,Throat |  | 0 | 0 | n/a |
| Chronic Specialty: Unstable-Eye |  | 108 (17.2%) | 102 (16.2%) | 0.03 |
| Dermatologic |  | 113 (18.0%) | 107 (17.0%) | 0.03 |
| Injuries/Adverse Effects: Minor |  | 197 (31.3%) | 199 (31.6%) | 0.01 |
| Injuries/Adverse Effects: Major |  | 205 (32.6%) | 214 (34.0%) | 0.03 |
| Psychosocial: Time Limited, Minor |  | 65 (10.3%) | 53 (8.4%) | 0.07 |
| Psychosocial:Recurrent or Persistent,Stable |  | 197 (31.3%) | 211 (33.5%) | 0.05 |
| Psychosocial:Recurrent or Persistent,Unstable |  | 112 (17.8%) | 134 (21.3%) | 0.09 |
| Signs/Symptoms: Minor |  | 481 (76.5%) | 490 (77.9%) | 0.03 |
| Signs/Symptoms: Uncertain |  | 547 (87.0%) | 535 (85.1%) | 0.06 |
| Signs/Symptoms: Major |  | 548 (87.1%) | 533 (84.7%) | 0.07 |
| Discretionary |  | 171 (27.2%) | 171 (27.2%) | 0 |
| See and Reassure |  | 51 (8.1%) | 44 (7.0%) | 0.04 |
| Prevention/Administrative |  | 348 (55.3%) | 363 (57.7%) | 0.05 |
