# Supplementary material for: Does early palliative identification improve the use of palliative care services?
Source: PLoS One. 2020 Jan 31;15(1):e0226597. doi: 10.1371/journal.pone.0226597 (PMC6994244; doi:10.1371/journal.pone.0226597)
Supplement: S8 Table — (DOCX) [file pone.0226597.s008.docx]

**S8 Table. Baseline characteristics of patients who remained alive by the end of follow-up period in the Intervention Group and the matched Control Group.**

| **VARIABLE** | **VALUE** | **Intervention Group**  **N=556** | **Control Group**  **N=556** | **Standardized difference** |
| --- | --- | --- | --- | --- |
| Sex | F | 263 (47.3%) | 253 (45.5%) | 0.04 |
|  | M | 293 (52.7%) | 303 (54.5%) | 0.04 |
| Age | Mean ± SD | 69.6 ± 14.0 | 68.7 ± 14.8 | 0.07 |
|  | Median (IQR) | 70 (60-80) | 69 (59-80) | 0.06 |
| Income quintile | 1-lowest | 111 (20.0%) | 106 (19.1%) | 0.02 |
|  | 2 | 102 (18.3%) | 107 (19.2%) | 0.02 |
|  | 3 | 112 (20.1%) | 114 (20.5%) | 0.01 |
|  | 4 | 103 (18.5%) | 108 (19.4%) | 0.02 |
|  | 5-highest | 128 (23.0%) | 121 (21.8%) | 0.03 |
| Rural resident | Y | 95 (17.1%) | 105 (18.9%) | 0.05 |
| Local Health Integration Network (LHIN) of residence | South West | < 6 | <6 | 0 |
|  | Hamilton Niagara Haldimand Brant | < 6 | <6 | 0 |
|  | Central West | 10 (1.8%) | 10 (1.8%) | 0 |
|  | Mississauga Halton | 8 (1.4%) | 8 (1.4%) | 0 |
|  | Toronto Central | 37 (6.7%) | 37 (6.7%) | 0 |
|  | Central | 51 (9.2%) | 51 (9.2%) | 0 |
|  | Central East | 39 (7.0%) | 39 (7.0%) | 0 |
|  | South East | 13 (2.3%) | 13 (2.3%) | 0 |
|  | Champlain | 278 (50.0%) | 278 (50.0%) | 0 |
|  | North Simcoe Muskoka | 115 (20.7%) | 115 (20.7%) | 0 |
|  | North East | < 6 | <6 | 0 |
| ***Pre-existing health problems and resource utilization in the 2 years before the Index date*** |  |  |  |  |
| Resource utilization band | 0-3 | 123 (22.1%) | 126 (22.7%) | 0.01 |
|  | 4 | 171 (30.8%) | 149 (26.8%) | 0.09 |
|  | 5 | 262 (47.1%) | 281 (50.5%) | 0.07 |
| Aggregated Diagnostic Groups (ADG) score | Mean ± SD | 8.8 ± 3.5 | 8.8 ± 3.4 | 0.02 |
|  | Median (IQR) | 9 (6-11) | 9 (6-11) | 0.03 |
|  | 0-5 | 101 (18.2%) | 100 (18.0%) | 0 |
|  | 6-7 | 105 (18.9%) | 103 (18.5%) | 0.01 |
|  | 8-9 | 123 (22.1%) | 111 (20.0%) | 0.05 |
|  | 10-11 | 112 (20.1%) | 125 (22.5%) | 0.06 |
|  | >=12 | 115 (20.7%) | 117 (21.0%) | 0.01 |
| Time Limited: Minor |  | 161 (29.0%) | 164 (29.5%) | 0.01 |
| Time Limited: Minor-Primary Infections |  | 297 (53.4%) | 294 (52.9%) | 0.01 |
| Time Limited: Major |  | 166 (29.9%) | 183 (32.9%) | 0.07 |
| Time Limited: Major-Primary Infections |  | 111 (20.0%) | 117 (21.0%) | 0.03 |
| Allergies |  | 27 (4.9%) | 26 (4.7%) | 0.01 |
| Asthma |  | 60 (10.8%) | 59 (10.6%) | 0.01 |
| Likely to Recur: Discrete |  | 283 (50.9%) | 275 (49.5%) | 0.03 |
| Likely to Recur: Discrete-Infections |  | 133 (23.9%) | 137 (24.6%) | 0.02 |
| Likely to Recur: Progressive |  | 113 (20.3%) | 112 (20.1%) | 0 |
| Chronic Medical: Stable |  | 410 (73.7%) | 440 (79.1%) | 0.13 |
| Chronic Medical: Unstable |  | 330 (59.4%) | 321 (57.7%) | 0.03 |
| Chronic Specialty: Stable-Orthopedic |  | 15 (2.7%) | 17 (3.1%) | 0.02 |
| Chronic Specialty: Stable-Ear,Nose,Throat |  | 32 (5.8%) | 27 (4.9%) | 0.04 |
| Chronic Specialty: Unstable-Orthopedic |  | 114 (20.5%) | 102 (18.3%) | 0.05 |
| Chronic Specialty: Unstable-Ear,Nose,Throat |  | 29 (5.2%) | 31 (5.6%) | 0.02 |
| Chronic Specialty: Unstable-Eye |  | 102 (18.3%) | 93 (16.7%) | 0.04 |
| Dermatologic |  | 139 (25.0%) | 119 (21.4%) | 0.09 |
| Injuries/Adverse Effects: Minor |  | 146 (26.3%) | 153 (27.5%) | 0.03 |
| Injuries/Adverse Effects: Major |  | 171 (30.8%) | 175 (31.5%) | 0.02 |
| Psychosocial: Time Limited, Minor |  | 40 (7.2%) | 45 (8.1%) | 0.03 |
| Psychosocial:Recurrent or Persistent, Stable |  | 173 (31.1%) | 185 (33.3%) | 0.05 |
| Psychosocial:Recurrent or Persistent,Unstable |  | 78 (14.0%) | 83 (14.9%) | 0.03 |
| Signs/Symptoms: Minor |  | 378 (68.0%) | 389 (70.0%) | 0.04 |
| Signs/Symptoms: Uncertain |  | 470 (84.5%) | 468 (84.2%) | 0.01 |
| Signs/Symptoms: Major |  | 447 (80.4%) | 436 (78.4%) | 0.05 |
| Discretionary |  | 148 (26.6%) | 157 (28.2%) | 0.04 |
| See and Reassure |  | 41 (7.4%) | 42 (7.6%) | 0.01 |
| Prevention/Administrative |  | 266 (47.8%) | 265 (47.7%) | 0 |
